# Promoting safe infant feeding practices – the importance of structural, social and contextual factors in Southern Africa

**DOI:** 10.7448/IAS.16.1.18037

**Published:** 2013-02-07

**Authors:** Ray Lazarus, Helen Struthers, Avy Violari

**Affiliations:** 1 Perinatal HIV Research Unit, University of the Witwatersrand/Chris Hani Baragwanath Hospital, Soweto, South Africa; 2 Anova Health Institute, Johannesburg, South Africa

**Keywords:** infant feeding, guidelines, prevention, vertical transmission of HIV, exclusive breastfeeding, formula feeding, mixed feeding, counselling, community engagement

## Abstract

There has been significant progress towards the goal of eliminating vertical transmission of HIV by 2015. However, a question that remains is how we can most effectively prevent late postnatal transmission of HIV through infant feeding. Guidelines published by the World Health Organization in 2010 have been widely adopted. These guidelines place strong emphasis on exclusive breastfeeding, in some countries over-turning a prior emphasis on formula feeding. Where available, provision of antiretroviral treatment for HIV-positive mothers or prophylaxis for infants offers additional protection against vertical transmission through infant feeding. However, merely changing guidelines is not sufficient to change practice, particularly with regard to culturally sanctioned forms of feeding, such as mixed feeding. This commentary highlights structural, social and contextual barriers to effective implementation of the guidelines and suggests ways to address some of these barriers.

## Introduction

The latest report on the global AIDS epidemic [1] shows a decline of 24% between 2009 and 2011 in the rate of vertical transmission of HIV-1 in sub-Saharan Africa. These figures confirm significant progress towards achieving the United Nations’ goal of virtually eliminating new infections among children by 2015. In South Africa, for example, the national rate of infection amongst HIV-exposed infants at 4–8 weeks was 3.5% in 2010 [2], while preliminary figures for 2011 [3] indicate a national rate of 2.7%. The final South African vertical transmission rate for 2011, taking into account late post-natal transmission is not yet available. However, estimates of rates at 18 months are sobering, suggesting that the final overall rate for 2010 could be as high as 12–18% [4]. Since the primary vector for late post-natal transmission is infant feeding practice, increased attention in this regard is urgently needed [2].

The question remains: how to promote safe feeding practice most effectively? This commentary highlights the importance of looking beyond technical issues and taking into account structural, social and contextual factors. We draw mainly on the South African experience, which includes substantial research and debate on the issue.

## Discussion

### Preventing vertical transmission through infant feeding practices

International guidelines on infant feeding to prevent vertical transmission of HIV have changed numerous times in response to new research findings [5]. These shifts have been most evident in relation to formula- vs. exclusive breastfeeding. An early emphasis on breastfeeding was later replaced by a recommendation to promote informed choice between breastfeeding and formula-feeding, subject to it being acceptable, feasible, affordable, sustainable and safe (the AFASS criteria). The most recent changes have responded to two strands of research. First, in resource-constrained settings, balancing the risks of both HIV and other common infections, breastfeeding has been found to be generally safer than formula-feeding for infants’ *overall* health and survival [6]. Second, it has been shown that maternal treatment with highly active antiretroviral therapy (HAART) and/or infant antiretroviral prophylaxis significantly reduces postnatal HIV transmission through breastfeeding [7–12].

In line with the above findings, the World Health Organization (WHO) revised its guidelines on infant-feeding by HIV-positive mothers [6]. The new guidelines recommend breastfeeding exclusively for six months, then introducing complementary food, with continued breastfeeding until 12 months or until a nutritionally adequate and safe diet without breast milk can be provided. These guidelines have been widely accepted. The South African government, for example, aligned its policy with the guidelines, with the proviso that babies who test negative should receive appropriate antiretroviral prophylaxis relative to the treatment status of their mothers for as long as they continue to breastfeed [13–15]. There was also a shift in policy on formula-feeding, which was previously supported as an option to prevent vertical transmission. Although HIV-positive mothers may still elect to formula-feed, formula is no longer provided free except where medical conditions prevent breastfeeding [16].

However, changing infant feeding *practice* is more complex than simply changing guidelines [17]. As pointed out by Kippax [18], all prevention involves changing *social* practices; thus “effective prevention … requires that public health addresses people not only as individuals but also as connected members of groups, networks and collectives who interact (talk, negotiate, have sex, use drugs, etc.) together” (p. 6). Without such engagement, individual-level change will not be effectively sustained.

Infant feeding is clearly a social practice, involving not only the directly participating mother and baby but also the social context of that interaction. This includes many other role-players, for example, male partners and female relatives (particularly, older relatives such as mothers, mothers-in-law and aunts), who commonly exchange ‘lay knowledge’ [19] on the best way to feed babies. Moreover, as highlighted by Zachariah *et al*. [20], this context involves “gendered power relations within and beyond the household [that] are likely to have a key role in whether or not [and how] mothers … engage with PMTCT programmes” (p. 2), including infant feeding prescripts.

### Challenges to implementation

It is our contention that structural, social and contextual factors have not been given enough attention in the development and implementation of infant feeding guidelines. Failure in this respect may compromise gains from effective ante- and peri-natal prevention. A number of challenges are highlighted below.

#### ART coverage

Effective implementation of the guidelines requires high rates of coverage of extended antiretroviral treatment for the mother or prophylaxis for the infant, continuing beyond the immediate postpartum period. However, coverage is often variable, with failures occurring along each step of the prevention of mother-to-child transmission (PMTCT) cascade. As a result, significant numbers of mother–infant pairs do not receive effective antiretroviral prophylaxis/treatment, both antenatal and postpartum [4]. A recent South African report [2], for example, confirmed weaknesses at a number of points in the PMTCT cascade. Particularly worrying was the low rate of reported intention to obtain a PCR test for the infant at 4–6 weeks. This could well feed into low uptake of antiretroviral prophylaxis for breastfeeding infants who test negative at that point and whose mothers are not on HAART.

#### Adherence

Unlike randomized, controlled trials, where adherence is strongly promoted and closely supervised, in real-world application, the same degree of control is seldom possible [20]. So, even when there is adequate access to postnatal antiretroviral prophylaxis/treatment, without high levels of support from health workers and family, adherence to daily medication for the mother and/or baby may not be consistently maintained over the full extended period of breastfeeding recommended in the guidelines: that is, daily for at least six months and preferably 12 months.

#### Exclusive breastfeeding

Given operational challenges to consistent antiretroviral cover and to ensuring adherence, exclusivity in breastfeeding by HIV-positive women remains a critical safeguard. However, mixed feeding practices are near-universal [21] and, in Southern Africa, despite strong cultural pressures to breastfeed, it is not the norm to breastfeed *exclusively* [22–24]. Community surveys in South Africa have reported figures of 8% [25] and 25% [26] for exclusive breastfeeding, with the latter study showing a rate of 51.3% for mixed feeding (formula and other solid foods with breast milk). In a clinic-based study in 2010 [2], only 28% of all mothers and 20.4% of HIV-positive mothers reported exclusively breastfeeding in the last eight days, while 44.8% and 18.1%, respectively, reported mixed feeding. In a recent update of the study [3], 56.3% of HIV-positive mothers reported “non-mixed breastfeeding” and 43.7% reported mixed feeding of their HIV-exposed babies at four to eight weeks of age. While the latter figures suggest considerable improvement in the rates of exclusive breastfeeding, a substantial proportion of HIV-positive mothers continue to report high-risk infant feeding practices contrary to the 2010 guidelines.

#### Formula-feeding

In South Africa, a number of studies have reported fairly widespread use of formula in the general population, especially amongst HIV-positive mothers: 27.2% of all mothers in a clinic sample (62% of HIV-positive mothers) [2] and 22.5% of a community sample, with significant numbers buying their own formula) [26]. Thus, many still opt or are obliged to formula-feed – including not only HIV-positive mothers but also grannies or others responsible for the care of infants of teenage or working mothers – a pattern that may not be unique to South Africa. The shift away from formula-feeding as an optional recommendation may mean that these caregivers do not receive support to formula-feed appropriately and safely.

## Mixed feeding – responding to the reality on the ground

Although the WHO guidelines [6] note that, in the absence of antiretroviral prophylaxis, mixed feeding significantly increases the risk of HIV transmission, they do not provide specific guidance on how to address this widespread practice when antiretroviral prophylaxis is not assured. The South African guidelines [14] merely reiterate that mixed feeding should be discouraged. As with earlier guidelines recommending early and abrupt cessation of breastfeeding to reduce MTCT, the current recommendations, while medically sound, fail to address the lived experience of women trying to do the best they can for their babies [27].

The 2010 guidelines [6] do highlight the need for “skilled counselling and support in appropriate infant feeding practices … [for] all pregnant women and mothers” (p. 4). Likewise, the South African 2010 PMTCT evaluation report [2] recommends increasing rates of infant feeding counselling to improve feeding practices. However, these recommendations fail to take account of the fact that health workers have until fairly recently been required to promote formula-feeding. Many have thoroughly internalized the view that HIV-positive women should not breastfeed, a view also held by some HIV-positive women, their families and communities (R. Lazarus, H. Struthers, A. Violari, unpublished report). Health workers also differ in their emphases on one or another feeding option, perhaps as a result of their own bias, or one encouraged in a particular health facility [17, 28]. Without thorough reorientation, they are unlikely to correctly and consistently communicate complex information [17] that includes not only guidance on feeding practices but also their rationale and reasons for changing from previous guidance. Health workers may also be uncertain on how to respond to mothers who insist on formula-feeding to accommodate issues such as work or other reasons for absence from home.

Moreover, it is not just a question of increasing the rates of counselling but also addressing its quality [28, 29]. Infant feeding counselling involves more than just conveying “messages” – it requires being open to hearing from women. Women bring to the counselling encounter a range of understandings that influence how they adapt healthcare recommendations to fit their own circumstances [27, 30] and views prevalent in their communities. For example, mixed feeding may stem from beliefs that breast milk is not nutritious enough to satisfy infants even as young as a few weeks [24] or that supplementing breast milk with formula or other food, by reducing the amount of breast milk consumed by the infant, reduces the likelihood of HIV transmission [31]. The advice of health workers discouraging mixed feeding tends to be disregarded not only because it is often directive but also because it contradicts conventional wisdom and fails to engage with what women think and do. The consequence is that women's alternative practices such as very early supplementation remain largely hidden from health workers [24].

In any event, to expect counselling alone to resolve the issue of mixed feeding would be naive. Many mothers introduce liquids or semi-solids from a very young age [24, 26, 32]. In so doing, they are acting in terms of their own perceptions and understanding of what is best for their babies, as well as responding to normative pressures based on lay understandings of their immediate family and the community in which they are embedded [24, 32]. Factors such as economic dependence appear to influence how susceptible women are to social and family pressures regarding feeding practice [33–35] and working mothers face yet other constraints. Concerns about HIV-related stigma may also influence feeding practices. Adopting locally unusual feeding practices – such as strict avoidance of mixed feeding – may arouse suspicion about a mother's HIV status and expose her to stigmatization [32]. Stigma, whether anticipated or directly experienced, may thus encourage mixed feeding and the early introduction of complementary foods in line with local practices. These factors cannot be addressed solely by counselling, however good its quality.

These structural, social and contextual factors need to be taken into account, if changed guidelines on infant feeding are to be applied as intended and achieve their objective of reducing the risk of mother-to-child transmission of HIV, especially where access to antiretroviral prophylaxis and its regular daily use on a long-term basis cannot be assured.

## Recommendations

To avoid unintended consequences, it is essential that steps are taken to ensure programmatic support for changed policies on infant feeding. Every effort should be made to ensure adequate coverage of antiretroviral prophylaxis/treatment (ante-, peri- and post-natal); that infants are brought for PCR testing at six weeks; and that infants who test negative continue to receive appropriate antiretroviral prophylaxis, if their mothers are not on HAART. In this regard, planned changes in donor funding of HIV programmes in developing countries could compromise healthcare delivery [36], including with respect to coverage and retention along the PMTCT cascade. Where prophylaxis/treatment is available, it is all the more important to ensure careful follow-up to promote adherence over the full period of breastfeeding.

However, given operational challenges, it remains critical to promote exclusive breastfeeding amongst HIV-positive mothers and to discourage mixed feeding. To do so effectively means taking account of social and contextual factors.

Specific recommendations are, first, that general messages and top-down directives are not enough to change entrenched normative views in communities and amongst health workers about appropriate feeding practices for infants in general and especially those whose mothers are HIV-positive. A more open and sustained engagement is needed to facilitate dialogue about infant feeding with HIV-positive mothers, families and communities – and health workers. (In this regard, a recent special issue of this journal [37] provides a review and examples of good practice in community engagement.)

Second, this dialogue needs to acknowledge and engage with views on appropriate infant-feeding practices for HIV-positive women specifically. The view that HIV-positive women should not breastfeed (as per earlier prescripts) will not disappear overnight, especially given the complexity of the argument why, despite some risk of vertical transmission, breastfeeding is recommended. As part of this dialogue, there also needs to be frank acknowledgement of the apparent contradictions in the various changes in infant feeding guidelines and a willingness to explain the rationale for the latest guidelines.

Third, since feeding intentions and practices are influenced by and negotiated between the mother and significant others, there is a need to understand the ways in which women and their families interpret guidance on exclusive breastfeeding and, particularly, mixed feeding. This should include exploring and engaging with normative views about babies’ nutritional needs and the adequacy of breast milk to meet those needs, as well as patterns of feeding and sleeping (such as quantity, frequency and duration), as these appear to contribute to the early supplementation of breast milk (and formula) [24] (R. Lazarus, H. Struthers, A. Violari, unpublished report).

Fourth, for various reasons, a substantial number of women may continue to use formula. It is important to encourage non-judgmental discussion of this choice to avoid a situation where women feel obliged to hide their feeding practice (which may well include mixing formula- and breastfeeding) from health workers and cannot therefore be helped to use formula safely.

Finally, given a widely identified pattern of inadequate counselling practice, it is essential to develop a broader, more open-ended and responsive approach in infant feeding counselling. This will require devoting more resources to training health workers, specifically counsellors; ensuring the necessary preconditions for effective counselling such as time, space and mentoring; and, where possible, extending the use of promising peer counsellor models [24, 38, 39].

## Conclusions

It is clear that guidelines are never implemented exactly as prescribed but are inevitably adapted to social environments and modified in the face of competing views. As Desclaux *et al*. [40] note, “overarching scientifically grounded prevention and care models are always social technologies” (p. 804). Hence, as Gray and Saloojee [41] argue, rather than expecting that caregivers and mothers will necessarily follow the dictates of experts, strategies to prevent postnatal transmission of HIV must be designed around their needs and realities. Acknowledging the multiplicity of factors that affect implementation and engaging with the opinions and differing circumstances of HIV-positive women, their families and communities, rather than being idealistic, is thus the only realistic approach to promoting safer infant feeding.
